# Lung function, symptoms and inflammation during exacerbations of non-cystic fibrosis bronchiectasis: a prospective observational cohort study

**DOI:** 10.1186/s12931-015-0167-9

**Published:** 2015-02-07

**Authors:** Simon E Brill, Anant RC Patel, Richa Singh, Alexander J Mackay, Jeremy S Brown, John R Hurst

**Affiliations:** UCL Respiratory Medicine, University College London Medical School, Royal Free Campus, Rowland Hill Street, London, NW3 2PF UK

**Keywords:** Bronchiectasis, Peak expiratory flow rate, Health-related Quality of Life, Respiratory questionnaire, Inflammation

## Abstract

**Background:**

Exacerbations of non-cystic fibrosis bronchiectasis cause significant morbidity but there are few detailed data on their clinical course and associated physiological changes. The biology of an exacerbation has not been previously described.

The purpose of this study was to describe changes in lung function, symptoms, health status and inflammation during the development and recovery from community-treated exacerbations.

**Methods:**

This was a prospective observational cohort study of 32 outpatients with non-cystic fibrosis bronchiectasis conducted between August 2010 and August 2012. Patients completed a symptom diary card and measured their peak expiratory flow rate (PEFR) daily. Exacerbations were defined as oral antibiotic treatment taken for a worsening of respiratory symptoms. Symptoms and peak flow at exacerbation were analysed, and further measurements including the COPD Assessment Test (CAT) and inflammatory markers were also compared to baseline values.

**Results:**

At baseline, health status was significantly related to lung function, prognostic severity and systemic inflammation. 51 exacerbations occurred in 22 patients. Exacerbation symptoms began a median (interquartile range) of 4 (2, 7) days before treatment started and the median exacerbation duration was 16 (10, 29) days. 16% had not recovered by 35 days. At exacerbation, mean PEFR dropped by 10.6% (95% confidence interval 6.9-14.2, p < 0.001) and mean CAT score increased by 6.3 units (3.6-9.1, p = 0.001), median symptom count by 4 (2.25, 6, p < 0.001), and mean CRP by 9.0mg/L (2.3-15.8, p = 0.011). Exacerbations where PEFR fell by ≥10% were longer with more symptoms at onset.

**Conclusion:**

Exacerbations of non-CF bronchiectasis are inflammatory events, with worsened symptoms, lung function and health status, and a prolonged recovery period. Symptom diary cards, PEFR and CAT scores are responsive to changes at exacerbation and may be useful tools for their detection and monitoring.

**Electronic supplementary material:**

The online version of this article (doi:10.1186/s12931-015-0167-9) contains supplementary material, which is available to authorized users.

## Background

Non-cystic fibrosis (CF) bronchiectasis is a chronic lung disease characterised by irreversibly damaged and dilated airways leading to recurrent episodes of bronchial sepsis (‘exacerbations’) [1]. Estimates from the United States suggest a prevalence of 52.3 cases per 100,000 [2], and mortality appears to be increasing by 3% annually [3]. The economic burden is high [2], largely due to recurrent exacerbations.

There has been growing interest in non-CF bronchiectasis, which has historically suffered from a lack of high-quality research [4]. Exacerbations cause significant morbidity and may also accelerate disease progression; recent clinical trials have therefore focused on strategies to prevent them [5,6]. However, changes in lung function and symptomatology during their development and recovery - especially community-treated exacerbations - have not been previously described. These data are vital to develop improved disease monitoring tools or surrogate trial endpoints.

To address this lack of data we have investigated changes in lung function, symptoms, health status and inflammation before, during and after community-treated exacerbations in patients with non-CF bronchiectasis. Some preliminary analysis has previously been presented in abstract form [7].

## Methods

### Study design

This was a prospective, observational cohort study conducted using outpatients at the Royal Free Hospital, London, United Kingdom. Ethical approval was obtained (reference 10/H0720/43) and all patients provided written, informed consent.

### Recruitment and stable visits

Patients with a clinical diagnosis of bronchiectasis were identified from general respiratory outpatient clinics. At screening, bronchiectasis was confirmed on previous imaging. Aetiology was determined according to British Thoracic Society guidance [1] and recorded as idiopathic if no cause was found after investigation. Spirometry was performed in accordance with ATS/ERS guidance [8] using a Vitalograph Gold Standard spirometer (Vitalograph Ltd., Maids Morton, UK). Blood was collected for analysis of inflammatory markers. Patients also completed the Chronic Obstructive Pulmonary Disease (COPD) Assessment Test (CAT) [9] and St George’s Respiratory Questionnaire (SGRQ) [10]. CT images were independently assessed using the Bhalla score [11] by two independent observers (JAH and JRH) and differences resolved by consensus. Prognostic disease severity was calculated using the Bronchiectasis Severity Index (BSI) [12] online calculator (www.bronchiectasisseverity.com).

Once enrolled, patients monitored their morning post-medication peak expiratory flow rate (PEFR) each day, recording the best of three attempts (using a Mini-Wright peak flow meter, Clement Clarke International Ltd, Harlow, UK). They also recorded the presence or worsening (if the symptom was usually present) of up to 15 symptoms and any changes in their treatment on daily diary cards. The diary card, available in the online data supplement (Additional file 1), was designed to reflect the breadth of symptoms that may occur during bronchiectasis exacerbations; all patients received careful training at their first and subsequent appointments. After recruitment, patients attended six-monthly when stable and exacerbation-free for the preceding four weeks. These and the recruitment visits provided baseline data for comparison.

### Exacerbation visits

Patients with worsening symptoms attended the research clinic where they were assessed by a doctor specialised in respiratory medicine (SEB/ARCP/RS/AJM/JRH) and received treatment for exacerbation as needed. If an exacerbation was confirmed clinically, further blood was sampled, spirometry performed and the CAT administered. Patients who were unable to attend the clinic, or who did not notify the study team at the time of exacerbation, recorded their symptoms, PEFR, and the dates and type of any antibiotic therapy taken on their daily diary cards.

### Sample analysis

Blood was analysed for C-reactive protein (CRP) using a Modular Analytics E170 Module (Roche, Burgess Hill, UK) with detection limit 1 mg/L, plasma fibrinogen using the Clauss method (IL ACL Top Coagulation Analyzer; Instrumentation Laboratories, Lexington, MA), and white cell and neutrophil counts using an automated XE-2100 analyser (Sysmex Corp, Kobe, Japan). Serum was stored at −80°C and batch analysed for interleukin (IL)-6 using commercial sandwich ELISA kits (RD Systems, Abingdon, UK).

### Statistical analysis

The first two weeks of PEFR data following study enrolment were discarded to allow for the development of a monitoring routine. Following this, baseline stable PEFR for each patient was calculated as the mean of the first 14 days of diary card completion without worsening of any symptoms and subsequent PEFR values were converted to % stable for all analyses and reporting. Baseline day-to-day variability over the same period was calculated as % of recent best using the formula [(highest PEFR – lowest PEFR)/highest PEFR]* 100 [13].

Exacerbation was defined as oral antibiotic treatment taken for worsening chest symptoms, without hospital admission, as reported by the patient or administered by the study team. Day 0 (exacerbation onset) was defined as the first day of antibiotic therapy; data from the preceding two weeks and subsequent five weeks were included for analysis. Only exacerbations treated with antibiotics were included and this was therefore a healthcare utilisation definition of exacerbation.

To examine the length of treated exacerbations within this period, symptomatic onset was defined as the first of ≥2 consecutive days between day −14 and day 0 where ≥2 extra symptoms were recorded on the diary cards with no further symptom-free days between that day and day 0. Symptomatic recovery was defined as the first of ≥2 symptom-free days, or (if there was continuous symptom recording) when the number of symptoms returned to the pre-exacerbation count.

Data were tested for Normality using the Kolmogorov-Smirnov test and reported as median (interquartile range [IQR]) or mean (standard deviation [SD]) as appropriate. Means were compared using paired or independent t-tests as appropriate and medians were compared using the Wilcoxon signed-rank test for related samples or the independent-samples median test as appropriate. Relationships between variables were assessed using Pearson's correlation coefficient. Missing data were not imputed. SPSS Statistics version 21.0 (SPSS Inc) was used for analysis and results were deemed statistically significant at the 5% level (p values of <0.05).

## Results

**Figure 1:**
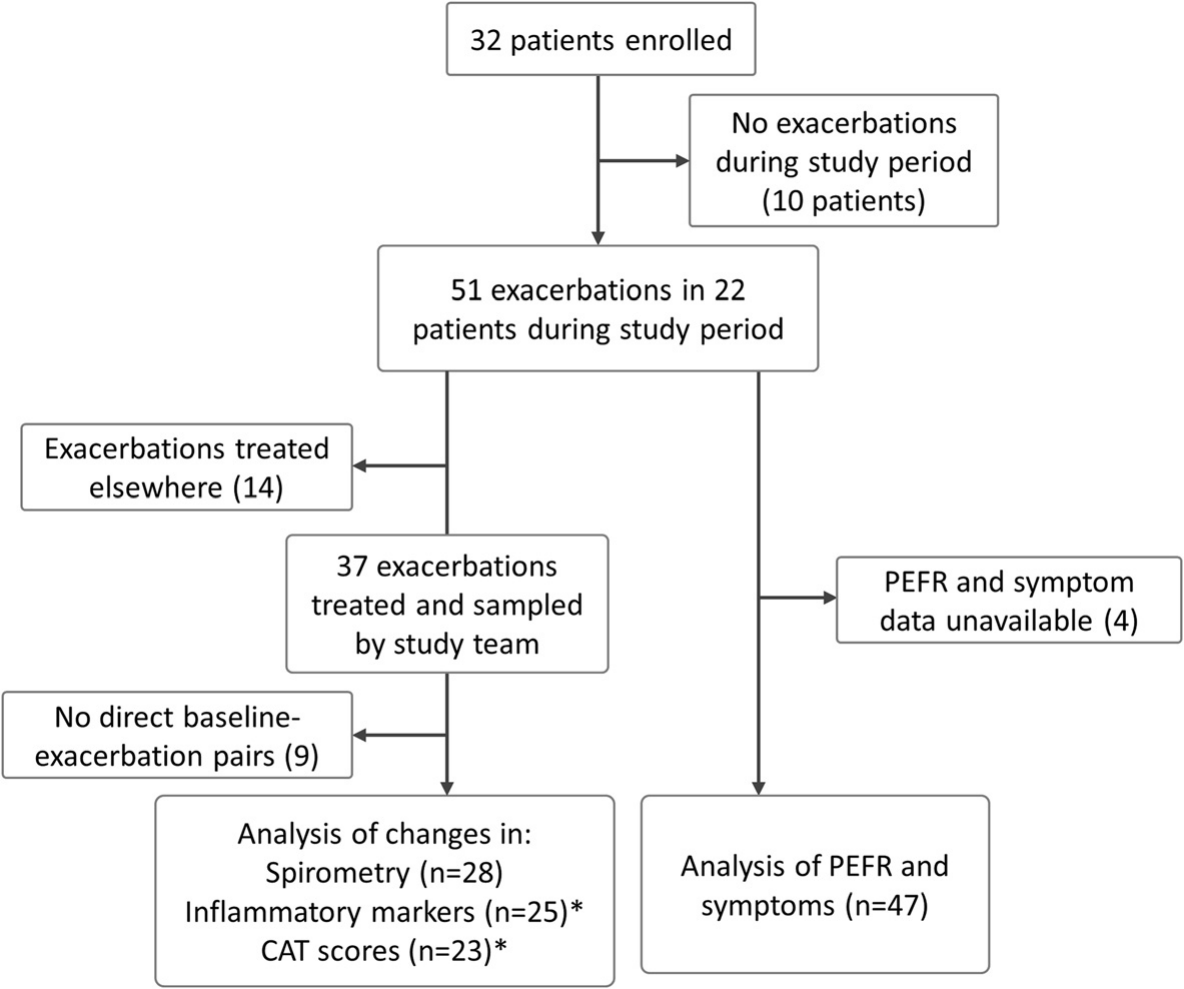
**Data flow for this study.** *Missing baseline-exacerbation pairs were due to a lack of baseline visits between exacerbations for comparison; further numbers variation is due to missing investigational data.

32 patients, enrolled between August 2010 and August 2012, were included for analysis. The median follow-up duration was 491 days. 51 exacerbations occurred in 22 of these patients during the study period with a median (IQR) of 1 (1, 3) per patient. Figure 1 illustrates the data flow for this study.

### Patient characteristics at baseline

Patient and disease characteristics at recruitment are reported in Table 1. Most patients were female, with a mean age of 61 years, with mainly post-infectious or idiopathic disease, typical for a European secondary care cohort.

**Table 1:** **Characteristics at study enrolment of all 32 patients with non-CF bronchiectasis and of the subset of 22 who experienced exacerbations during the study period**

	**Whole cohort (n = 32)**	**Patients experiencing exacerbations during study period (n = 22)**
Age (years), mean (SD)	61 (12)	59 (12)
Female patients, n (%)	27 (84)	19 (86)
Duration of follow up (days), median (IQR)	491 (386,603)	506 (390,648)
Ever smokers, n (%)	10 (31)	6 (27)
Aetiology, n (%)	Post-infectious: 16 (50)	Post-infectious: 13 (59)
	Idiopathic: 11 (34)	Idiopathic: 5 (23)
	Rheumatological: 2 (6)	Rheumatological: 1 (4)
	Other: 3 (9)	Other: 3 (14)
Bhalla score, mean (SD)	12 (9)	12 (9)
Number of lobes involved, median (IQR)	3.5 (2,5)	3.5 (2,5)
BSI score, median (IQR)	8.5 (4,12.8)	9 (4.5,11.3)
Severity (BSI classification), n (%)	Mild: 9 (28)	Mild: 6 (27)
	Moderate: 8 (22)	Moderate: 4 (18)
	Severe: 15 (50)	Severe: 12 (55)
Self-reported exacerbation frequency in previous year, median (IQR)	3 (2,5.8)	3 (2.3,6)
Asthma diagnosis, n (%)	7 (22)	6 (27)
Previous pseudomonas on sputum culture, n (%)	7 (22)	5 (23)
Previous hospitalisation, n (%)	9 (28)	8 (36)
Treatment details (all given as n (%))		
Short-acting inhaled bronchodilator	13 (41)	10 (45)
Long-acting inhaled bronchodilator	12 (38)	8 (36)
Inhaled corticosteroid use	16 (50)	11 (50)
Long term macrolide therapy, n (%)	2 (6)	2 (9)
Lung function measurements: (all given as mean (SD))		
FEV_1_(L), mean (SD)	1.96 (0.90)	2.02 (0.95)
FEV_1_% predicted (SD)	78 (26)	76 (28)
FEV_1_/FVC ratio, (SD)	0.71 (0.15)	0.71 (0.17)
Stable PEFR (L/min), (SD)	334 (104)	337 (110)
PEFR variability, % best (see text)	12.4 (8.2)	13.8 (9.4)
SGRQ and CAT scores (all given as mean (SD))		
Symptoms	48.8 (24.3)	50.2 (26.1)
Activity	40.3 (27.8)	41.8 (27.7)
Impact	27.2 (16.5)	29.4 (14.9)
Total SGRQ score	34.8 (18.1)	37.6 (16.9)
CAT score	16.6 (7.0)	17.2 (7.1)

As expected, there were significant relationships between health status and the physiological, prognostic and inflammatory severity of bronchiectasis. SGRQ scores were significantly correlated with FEV_1_ % predicted (r = −0.507, p = 0.003), BSI scores (r = 0.587, p < 0.001), and the serum inflammatory markers CRP [r = 0.390, p = 0.03], fibrinogen [r = 0.460, p = 0.013], and log[IL-6] [r = 0.480, p = 0.044]). CAT scores were closely related to SGRQ scores (r = 0.799, p < 0.001) and also significantly related to FEV_1_, (r = −0.624, p < 0.001), BSI scores (r = 0.493, p = 0.004) and inflammation (fibrinogen, r = 0.455, p = 0.013). No differences in baseline characteristics were detected between patients who suffered an exacerbation during the study period and those who did not.

Mean day-to-day PEFR variability in these patients was 12.4% (8.2), and there was no significant difference in patients with asthma (n = 6, 19%) compared to those without.

### Exacerbations

**Figure 2:**
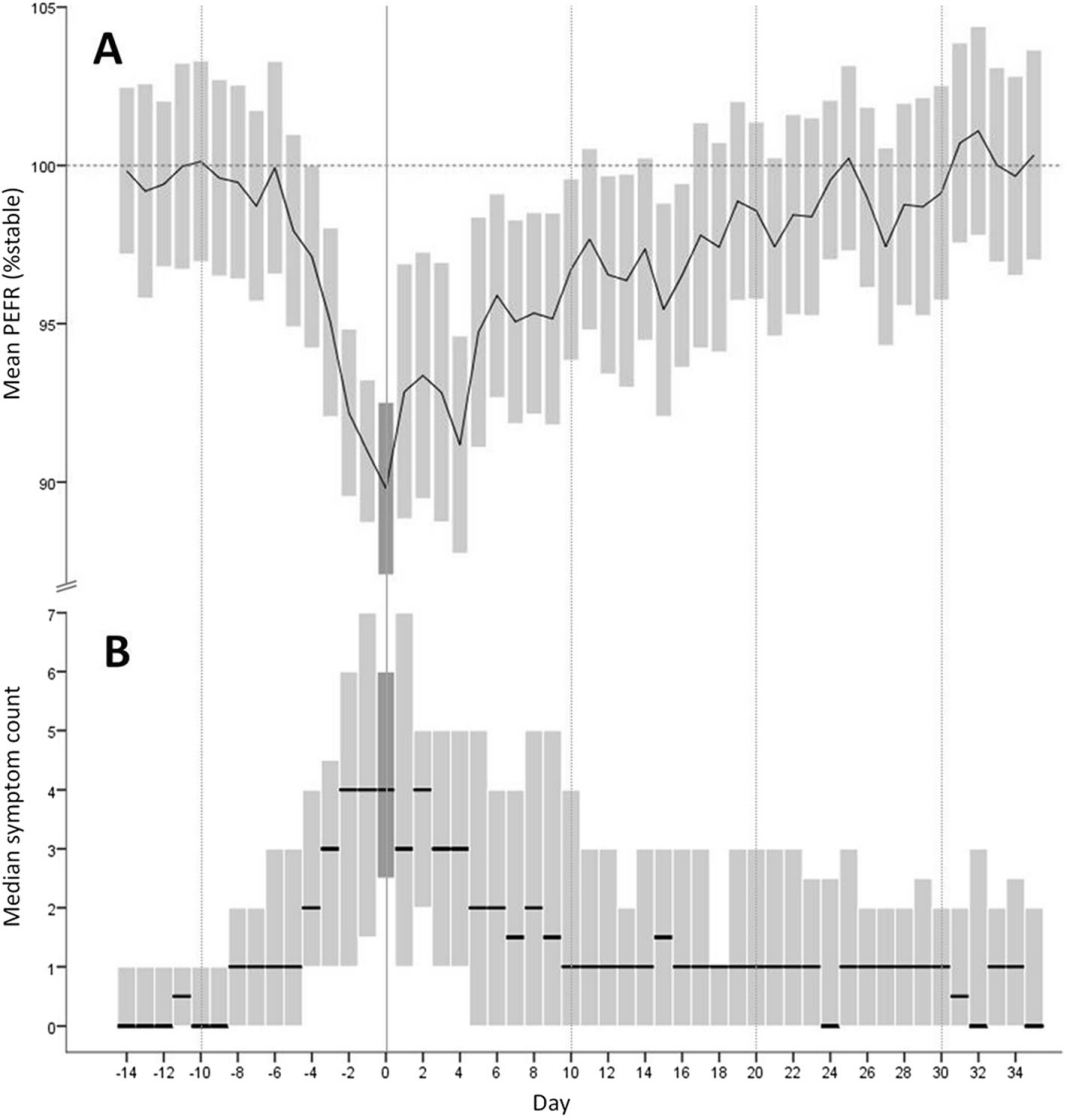
**Mean PEFR (A) and median diary card symptom count (B) before, during and after treated exacerbations of non-CF bronchiectasis.** Day 0 (highlighted) was the first day of antibiotic treatment. The bars represent 95% confidence intervals **(A)** and interquartile ranges **(B)**.

21 of the 22 patients who experienced exacerbations recorded PEFR and symptoms during 47 events, with data available for 82% and 78% of days respectively. The time-course of the changes in PEFR and symptoms during exacerbation onset and recovery is illustrated in Figure 2. All exacerbations received oral antibiotic therapy with a minimum duration of seven days.

### Changes in physiology with exacerbation

The mean PEFR first began to fall 6 days before treatment onset (day −6), becoming statistically significant at day −3 (4.9% reduction, 95% CI 1.6-8.2, p = 0.005 for comparison with day −14). The lowest PEFR was recorded on day 0, with a 10.6% reduction from stable (95% CI 6.9-14.2, p < 0.001), corresponding to a mean absolute reduction of 31 (19–42) L/min (p < 0.001). The mean PEFR returned to pre-exacerbation values at day 25, although by day 11 the difference was no longer statistically significant. Importantly, there was no significant difference in mean PEFR at day 0 in patients with a comorbid asthma diagnosis (11 exacerbations) compared to those without (93.4% (7.1) vs 88.5 (8.9), p = 0.112), and the PEFR reduction at exacerbation was not related to baseline PEFR variability. In community treated exacerbations there is therefore a statistically significant reduction in PEFR from at least three days before until at least eleven days following treatment onset.

In those exacerbations that were sampled and where baseline-exacerbation pairs were available (n = 28, in 20 patients), there was also a moderate reduction in FEV_1_ at exacerbation compared to previous baseline, both in absolute (90ml, 95% CI 15–164, p = 0.019) and % stable (4.1%, 95% CI 0.6-7.5, p = 0.022) values.

### Changes in symptoms with exacerbation

**Figure 3:**
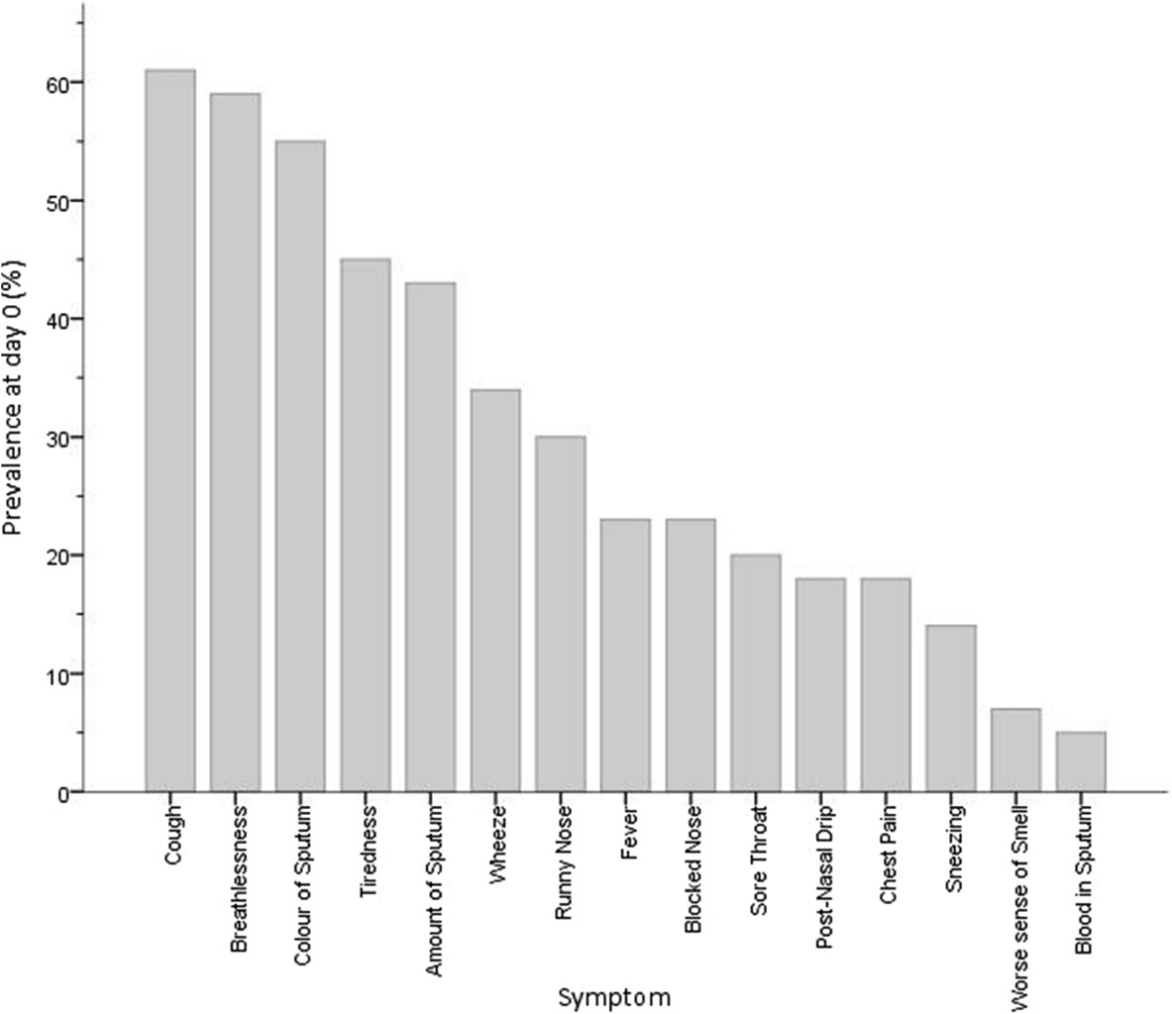
**Prevalence of reported symptoms at the onset of antibiotic treatment for exacerbations of non-CF bronchiectasis (n = 47).**

The number of reported daily symptoms increased from a median (IQR) of 0 (0, 1) at day −14 to 4 (2.25, 6 [p < 0.001]) at treatment onset. The most prevalent symptoms reported at exacerbation were increased cough (61%), breathlessness (59%) and change in sputum colour (55%). Figure 3 illustrates the prevalence of each of the 15 recorded symptoms at exacerbation onset.

**Figure 4:**
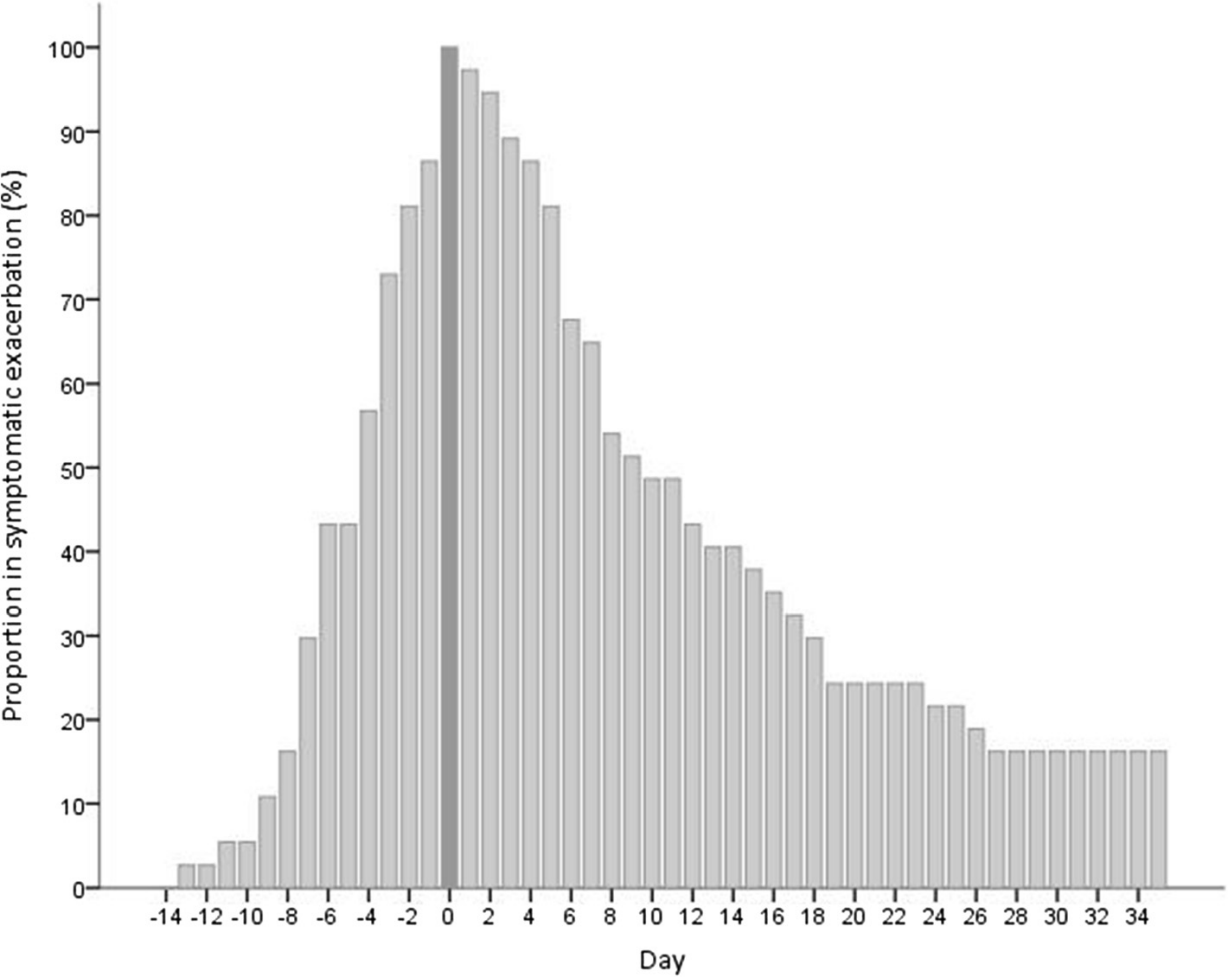
**Proportion of 37 exacerbations meeting symptomatic criteria for exacerbation by day.** Day 0 was the first day of treatment (highlighted). 16% of exacerbations had not recovered by day 35 based on failure to return to baseline on diary card symptom recording.

Using the definition detailed above, symptomatic exacerbation onset and recovery could be defined in 37 exacerbations, with the remainder not recording a symptomatic increase. The median (IQR) onset was 4 (7,2) days before treatment, and median overall exacerbation length 16 (10,29) days. There was a significant correlation between exacerbation length and the number of symptoms at treatment onset (r = 0.458, p = 0.004). Although the median recovery time after treatment start was 9 (5,20.5) days, 6 exacerbations (16%) had still not recovered symptomatically at 35 days after treatment onset. Figure 4 illustrates the proportion of these patients meeting symptomatic exacerbation criteria on each day of the time-course.

### Changes in health status with exacerbation

Paired CAT scores were available at exacerbation and preceding baseline from 23 exacerbations in 17 patients. At exacerbation, there was a mean (SD) increase of 6.3 (6.3) units over baseline (95% CI 3.6-9.1, p < 0.001).

### Relationship between changes in PEFR, symptoms and health status

Greater falls in PEFR at exacerbation onset were associated with greater symptom burden and prolonged recovery. In 14 exacerbations where PEFR fell by ≥10%, compared to 17 in which it did not, there was a higher median (IQR) day 0 symptom count (3 [1.5, 4.5] vs 5.5 [5,8], p = 0.009), and longer median (IQR) symptomatically defined exacerbation period (10 days [8,14] vs 17 [13, 28], p = 0.047).

There was also a significant correlation between the change in CAT score at exacerbation and the number of symptoms at exacerbation onset (r = 0.455, p = 0.038).

### Changes in systemic inflammatory markers with exacerbation

Table 2 reports the systemic biomarker results where paired samples were available at exacerbation onset and preceding baseline review. Serum fibrinogen, CRP and IL-6 all increased significantly at exacerbation. Interestingly the delay between symptom onset and treatment was weakly correlated to inflammatory change at exacerbation (rise in CRP; r = 0.469, p = 0.049) implying that delayed presentation associates with greater inflammation.

**Table 2:** **Serum inflammatory markers at baseline and exacerbation in non-CF bronchiectasis**

	**Baseline (Mean, SD)**	**Exacerbation (Mean, SD)**	**Number of exacerbations (Number of patients occurred in)**	**P-value for comparison**
White cell count (x10^9^/L)	6.8 (2.5)	7.3 (2.5)	25 (18)	0.351
Neutrophil count (x10^9^/L)	4.2 (1.9)	4.5 (1.7)	23 (18)	0.538
Fibrinogen (g/L)	3.6 (0.5)	4.1 (0.9)	20 (16)	0.001
CRP (mg/L)	3.0 (2.0)	12.0 (16.7)	24 (18)	0.011
IL-6 (log_10_ pg/mL)	0.65 (0.48)	0.97 (0.51)	22 (15)	0.038

## Discussion

This is the first study to conduct a detailed, prospective assessment of changes in symptoms and physiology prior to, at onset, and through the recovery of exacerbations in patients with non-CF bronchiectasis.

The key messages may be summarised thus. First, lung function and symptoms deteriorate between three and four days prior to patients initiating therapy, suggesting a window for earlier detection and treatment of exacerbations. A larger fall in PEFR was associated with greater symptom burden at exacerbation onset and a longer symptom recovery time. Second, the overall burden of these community exacerbations is high: the median symptom duration was 16 days, with lung function statistically abnormal for two weeks. Third, these community treated exacerbations were associated with a measurable systemic inflammatory response. Finally, the CAT reflects health status in the stable state and is responsive to changes at exacerbation in patients with bronchiectasis.

PEFR is a simple and well-established tool for assessing day-to-day changes in lung function. Patients learn the technique easily, the device is cheap and daily measurements are not unduly burdensome. Although spirometry is the most widely used measure of lung function in bronchiectasis, it requires more training and equipment and is not sufficiently responsive to changes with treatment [13]; newer techniques include the Lung Clearance Index (LCI) [14], although this requires still more specialist equipment and has yet to be evaluated in monitoring treatment response. In principle, PEFR therefore remains an attractive technique for disease self-monitoring.

We have shown a reduction of >10%, or 30L/min, in PEFR during these community-treated exacerbations. Full recovery did not occur until 25 days after treatment initiation. We have also shown that the reduction in PEFR at exacerbation is larger, and more closely related to reported symptoms and exacerbation duration, than FEV_1_. In addition, where there was a large change in PEFR, patients experienced worse health status, more symptoms and longer exacerbations. These data suggest that, where large changes are present, PEFR may be an effective surrogate marker for important clinical outcomes. However, changes below the level of day-to-day variability will be difficult to detect in individuals, particularly asthmatics, and this limits the utility of PEFR in making treatment decisions. Nonetheless, we have shown that PEFR change is measurable across a population and this, combined with its simplicity, may still prove more useful than spirometry for studies looking to assess changes in lung function.

This study is the first to describe the symptomatic development and duration of treated exacerbations of non-CF bronchiectasis. At the time of planning this study, there were no widely accepted symptomatic criteria for diagnosing an exacerbation, although these have subsequently been published [1]. In order to reflect current clinical practice for these mild community-treated exacerbations the decision to treat with antibiotics was therefore based either on the clinical judgement of the treating clinician or on the patient taking their stand-by antibiotic courses without formal criteria. However, in order to examine the length of treated exacerbations, a symptomatic definition was needed. Criteria defined elsewhere [1] require all of systemic upset, increased sputum volume or viscosity and increased sputum purulence to be present prior to antibiotic therapy being prescribed. Applying these criteria to our dataset, a day of onset could only be accurately defined in 17/47 (36%) of exacerbations. This was partially because the diary cards did not include ‘viscosity’ as a specific symptom, and because individual clinical judgement was used by both patients and clinicians when taking the decision to start antibiotics. Our simpler criteria, using two or more symptoms recorded on the diary cards over two or more days, were useful for detecting the symptomatic exacerbation onset and recovery of treated exacerbations, although we have not examined the utility of this definition in making treatment decisions. Surprisingly, increased cough was only recorded as a symptom in 60% of exacerbations, despite its prevalence having been reported as high as 98% at diagnosis [15]. The reason behind this is unclear, but may be a combination of reduced perception of a chronic symptom, under-reporting on the diary cards, or heterogeneity in the (non-standardised) indications for antibiotic therapy.

Patients waited on average four days after symptoms increased before they started therapy, and we found a weak but significant relationship between treatment delay and inflammatory change at exacerbation. This is potentially important, as it suggests that starting treatment early might reduce the severity and duration of the exacerbation. However, in some cases this delay may be appropriate, for example if the initial presentation is with viral upper respiratory tract symptoms for which antibiotic treatment would not necessarily be appropriate. The reasons underlying delays in treatment, and the clinical impact on exacerbation progression and recovery, therefore warrant further investigation.

In non-CF bronchiectasis, the SGRQ is currently the best validated measure of respiratory health status [10]. The CAT is an eight-item questionnaire, widely used as a simple and reliable measure of the impact of symptoms on health status [9] and the severity of exacerbations [16] in COPD, and is quick and simple to complete. We have shown here that the CAT is closely related to SGRQ score at baseline, with the strength of this relationship (r = 0.799) almost identical to that reported in COPD [9] and slightly stronger than previously reported in bronchiectasis [17], and also related to other important markers of lung function, disease severity and inflammation. Importantly, it was also sensitive to changes at exacerbations of bronchiectasis and correlated to exacerbation symptoms. The increase from baseline at bronchiectasis exacerbation was much higher than the reported minimum clinically important difference for COPD of 2 points [18]. The CAT may therefore be a simple method to monitor health status at baseline and exacerbation in bronchiectasis, and may be useful as a research tool for these patients.

Exacerbations of bronchiectasis are inflammatory events and heightened systemic inflammation has previously been shown, albeit in hospitalised patients compared to a separate group of patients who were not exacerbating [19]. For the first time, we have demonstrated increased systemic inflammation in the same patients between exacerbation and stable state in milder outpatient events. The largest rise at exacerbation, in CRP, crossed the usual upper limit of normal (5mg/dL) and related to symptoms at onset. CRP will be available to most researchers and may provide a useful marker to monitor inflammation during these events.

### Strengths of this study

This study provides accurate longitudinal information, prospectively collected, to track the development and recovery of exacerbation. This is in contrast to previous studies, which have been retrospective or compared exacerbation and stable state across different patients. In addition, the patients studied here were not hospitalised; their disease is mainly post-infectious or idiopathic, and therefore likely to be representative of the wider population of patients with bronchiectasis. The use of these simple clinical measurements in this population should be widely applicable to other researchers and clinicians in the field.

### Limitations of this study

Our data were observational and dependent on patients’ self-recording of measurements and exacerbation dates, as well as attending clinic for review when unwell. It is therefore possible that we did not detect all exacerbations that occurred within the study population, as well as being limited by other factors affecting observational research such as the non-standardisation of exacerbation treatment and missing data. As noted above, our definition of exacerbation was broad and based on the judgement of the patient or treating clinician. As such, some of these events may have not have met formal a priori criteria for exacerbation diagnosis had these been defined. In addition, our sample size was not large, particularly when looking at paired samples, and although it appears to represent a usual outpatient secondary care population selection bias cannot be excluded. Although these factors may have limited our ability to detect more subtle changes at exacerbation, they do not diminish the positive associations that we have reported.

## Conclusions

For the first time in non-CF bronchiectasis, we have described in detail the symptomatic and lung function changes during the development and recovery from exacerbation, and the relationships between lung function, exacerbation length, systemic inflammation and health status during exacerbations. We have shown that PEFR and symptom diary cards provide simple measures to define, track and monitor exacerbations, and that the CAT questionnaire may be a useful tool to monitor health status.

## Additional file

Additional file 1:
**The london bronchiectasis cohort patient symptom diary.**
