# Supplementary material for: Lung function, symptoms and inflammation during exacerbations of non-cystic fibrosis bronchiectasis: a prospective observational cohort study
Source: Respir Res. 2015 Feb 7;16(1):16. doi: 10.1186/s12931-015-0167-9 (PMC4324878; doi:10.1186/s12931-015-0167-9)
Supplement: Additional file 1: — The london bronchiectasis cohort patient symptom diary. [file 12931_2015_167_MOESM1_ESM.docx]

**ONLINE DATA SUPPLEMENT FOR:**

**Lung function, symptoms and inflammation during exacerbations of non-cystic fibrosis bronchiectasis: a prospective observational cohort study**

Simon E Brill, Anant RC Patel, Richa Singh, Alexander J Mackay, Jeremy S Brown, John R Hurst

Contains: sample copy of diary card document used in the study (February 2012)**
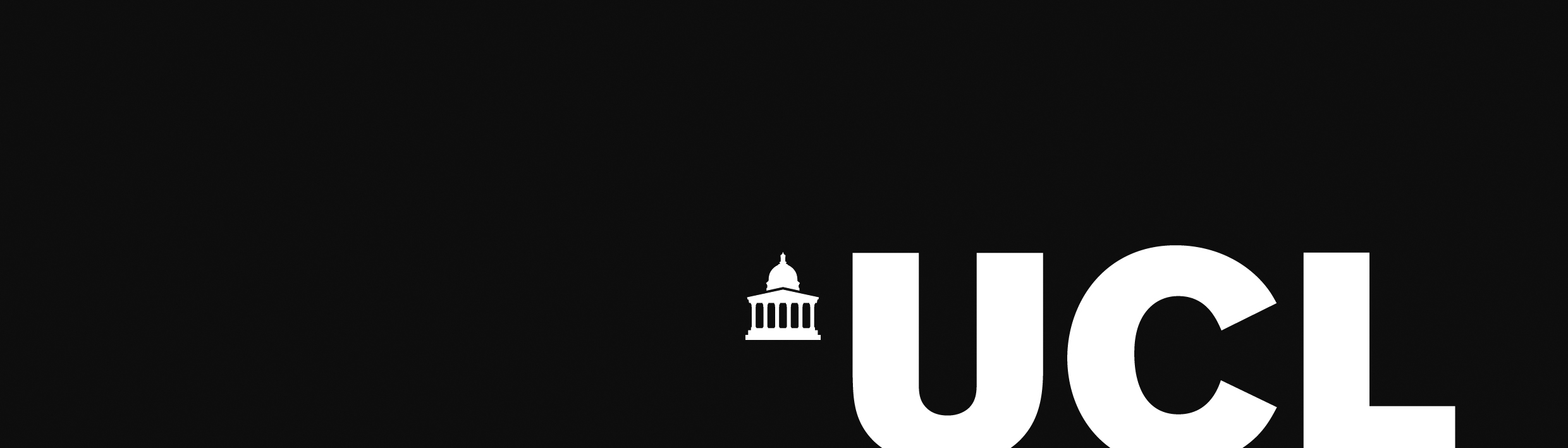
The London Bronchiectasis Cohort**

**Patient Symptom Diary**

**Feb 2012**

| **NAME** | | | |  | | | | | | | | | | | |  |  |  |  |  |  |
| --- | --- | --- | --- | --- | --- | --- | --- | --- | --- | --- | --- | --- | --- | --- | --- | --- | --- | --- | --- | --- | --- |
| **CODE / NUMBER** | | | |  | | | | | | B _ _ _ | | | | | |  |  |  |  |  |  |
| **NEXT APPOINTMENT** | | | |  | | | | | |  | | | | | |  |  |  |  |  |  |
|  |  |  | **TODAY my symptoms are the same as usual (tick)** | | **TODAY these symptoms are NEW or WORSE than usual** – tick all that apply. Please call us if you have worsening symptoms:  xxxxx-xxxxxx | | | | | | | | | | | | | | | | |
| Day | DATE | **Peak**  **Flow**  **TEST** |  |  | Breathlessness | Amount of Sputum | Colour of Sputum | Blood in Sputum | Cough | | Wheeze | Chest Pain | Tiredness | Fever | Sore Throat | | Runny Nose | Blocked Nose | Post-Nasal Drip | Sneezing | Worse sense of Smell |
| Wed | 1 |  |  | |  |  |  |  |  | |  |  |  |  |  | |  |  |  |  |  |
| Thu | 2 |  |  | |  |  |  |  |  | |  |  |  |  |  | |  |  |  |  |  |
| Fri | 3 |  |  | |  |  |  |  |  | |  |  |  |  |  | |  |  |  |  |  |
| Sat | 4 |  |  | |  |  |  |  |  | |  |  |  |  |  | |  |  |  |  |  |
| Sun | 5 |  |  | |  |  |  |  |  | |  |  |  |  |  | |  |  |  |  |  |
| Mon | 6 |  |  | |  |  |  |  |  | |  |  |  |  |  | |  |  |  |  |  |
| Tue | 7 |  |  | |  |  |  |  |  | |  |  |  |  |  | |  |  |  |  |  |
| Wed | 8 |  |  | |  |  |  |  |  | |  |  |  |  |  | |  |  |  |  |  |
| Thu | 9 |  |  | |  |  |  |  |  | |  |  |  |  |  | |  |  |  |  |  |
| Fri | 10 |  |  | |  |  |  |  |  | |  |  |  |  |  | |  |  |  |  |  |
| Sat | 11 |  |  | |  |  |  |  |  | |  |  |  |  |  | |  |  |  |  |  |
| Sun | 12 |  |  | |  |  |  |  |  | |  |  |  |  |  | |  |  |  |  |  |
| Mon | 13 |  |  | |  |  |  |  |  | |  |  |  |  |  | |  |  |  |  |  |
| Tue | 14 |  |  | |  |  |  |  |  | |  |  |  |  |  | |  |  |  |  |  |
| Wed | 15 |  |  | |  |  |  |  |  | |  |  |  |  |  | |  |  |  |  |  |
| Thu | 16 |  |  | |  |  |  |  |  | |  |  |  |  |  | |  |  |  |  |  |
| Fri | 17 |  |  | |  |  |  |  |  | |  |  |  |  |  | |  |  |  |  |  |
| Sat | 18 |  |  | |  |  |  |  |  | |  |  |  |  |  | |  |  |  |  |  |
| Sun | 19 |  |  | |  |  |  |  |  | |  |  |  |  |  | |  |  |  |  |  |
| Mon | 20 |  |  | |  |  |  |  |  | |  |  |  |  |  | |  |  |  |  |  |
| Tue | 21 |  |  | |  |  |  |  |  | |  |  |  |  |  | |  |  |  |  |  |
| Wed | 22 |  |  | |  |  |  |  |  | |  |  |  |  |  | |  |  |  |  |  |
| Thu | 23 |  |  | |  |  |  |  |  | |  |  |  |  |  | |  |  |  |  |  |
| Fri | 24 |  |  | |  |  |  |  |  | |  |  |  |  |  | |  |  |  |  |  |
| Sat | 25 |  |  | |  |  |  |  |  | |  |  |  |  |  | |  |  |  |  |  |
| Sun | 26 |  |  | |  |  |  |  |  | |  |  |  |  |  | |  |  |  |  |  |
| Mon | 27 |  |  | |  |  |  |  |  | |  |  |  |  |  | |  |  |  |  |  |
| Tue | 28 |  |  | |  |  |  |  |  | |  |  |  |  |  | |  |  |  |  |  |
| Wed | 29 |  |  | |  |  |  |  |  | |  |  |  |  |  | |  |  |  |  |  |
|  |  |  |  | |  |  |  |  |  | |  |  |  |  |  | |  |  |  |  |  |
|  |  |  |  | |  |  |  |  |  | |  |  |  |  |  | |  |  |  |  |  |

| Please record the dates of any additional antibiotics for your chest, or dates in hospital here: |
| --- |
